# New Method for Beat-to-Beat Fetal Heart Rate Measurement Using Doppler Ultrasound Signal

**DOI:** 10.3390/s20154079

**Published:** 2020-07-22

**Authors:** Tomasz Kupka, Adam Matonia, Michal Jezewski, Janusz Jezewski, Krzysztof Horoba, Janusz Wrobel, Robert Czabanski, Radek Martinek

**Affiliations:** 1 Łukasiewicz Research Network—Institute of Medical Technology and Equipment, PL41800 Zabrze, Poland; adamm@itam.zabrze.pl (A.M.); janusz.jezewski@itam.zabrze.pl (J.J.); krzysztof.horoba@itam.zabrze.pl (K.H.); januszw@itam.zabrze.pl (J.W.); 2 Department of Cybernetics, Nanotechnology and Data Processing, Silesian University of Technology, PL44100 Gliwice, Poland; michal.jezewski@polsl.pl (M.J.); robert.czabanski@polsl.pl (R.C.); 3 Department of Cybernetics and Biomedical Engineering, VSB—Technical University of Ostrava, 70800 Ostrava-Poruba, Czech Republic; radek.martinek@vsb.cz

**Keywords:** fetal monitoring, Doppler ultrasound signal, fetal heart rate, beat-to-beat variability

## Abstract

The most commonly used method of fetal monitoring is based on heart activity analysis. Computer-aided fetal monitoring system enables extraction of clinically important information hidden for visual interpretation—the instantaneous fetal heart rate (FHR) variability. Today’s fetal monitors are based on monitoring of mechanical activity of the fetal heart by means of Doppler ultrasound technique. The FHR is determined using autocorrelation methods, and thus it has a form of evenly spaced—every 250 ms—instantaneous measurements, where some of which are incorrect or duplicate. The parameters describing a beat-to-beat FHR variability calculated from such a signal show significant errors. The aim of our research was to develop new analysis methods that will both improve an accuracy of the FHR determination and provide FHR representation as time series of events. The study was carried out on simultaneously recorded (during labor) Doppler ultrasound signal and the reference direct fetal electrocardiogram Two subranges of Doppler bandwidths were separated to describe heart wall movements and valve motions. After reduction of signal complexity by determining the Doppler ultrasound envelope, the signal was analyzed to determine the FHR. The autocorrelation method supported by a trapezoidal prediction function was used. In the final stage, two different methods were developed to provide signal representation as time series of events: the first using correction of duplicate measurements and the second based on segmentation of instantaneous periodicity measurements. Thus, it ensured the mean heart interval measurement error of only 1.35 ms. In a case of beat-to-beat variability assessment the errors ranged from −1.9% to −10.1%. Comparing the obtained values to other published results clearly confirms that the new methods provides a higher accuracy of an interval measurement and a better reliability of the FHR variability estimation.

## 1. Introduction

Cardiotocography is a crucial part of modern perinatal medicine, which enables to monitor and assess a fetal condition during pregnancy and labor. It involves recording a fetal heart rate (FHR) signal against uterine contractile activity and fetal movements [1,2,3,4,5]. Normal fetal heart activity is an indirect evidence of adequate fetal oxygenation and preservation of the central nervous system functions [6]. The FHR signal values in the range 110 ÷ 150 bpm confirm a normal fetal heart activity. Accelerations or decelerations are defined as episodes of increasing or slowing of the heart rate. Another activity feature is an instantaneous variability, represented by two components: short-term variability (STV) describing FHR fluctuations occurring from beat-to-beat and long-term variability (LTV) defining periodic fluctuations in direction and extent of the STV.

The highest accuracy of the FHR signal determination is ensured by recording primary electrical excitations of a fetal heart, i.e., the fetal electrocardiogram (FECG) [7,8], which ensures very precise locations of characteristic points—R waves, representing subsequent fetal heartbeats. Based on time T_i_ between two consecutive R waves, the instantaneous FHR_i_ value is determined in beats per minute [bpm]:

(1) $${\mathrm{FHR}}_{i}=\frac{60,000}{T_{i}}$$

where: T_i_ is the interval between two consecutive heartbeats expressed in milliseconds.

Unfortunately, the use of fetal electrocardiography during pregnancy faces serious problems [9,10,11,12]. When acquiring an indirect FECG from maternal abdominal surface, its amplitude is much lower than the electrical activity from mother, and in addition, a high level of other interferences often prevents successful T_i_ measurement [13,14,15]. A good quality signal can be obtained invasively from an electrode directly attached to a fetal head, which can only be carried out during an advanced labor. These limitations caused that already in early 70s a Doppler ultrasound (US) method [16,17,18,19,20,21,22] was applied for monitoring of mechanical activity of the fetal heart. The Doppler echo signal reflected from moving heart is characterized by a complex structure originating from opening and closing of valves, as well as from movements of heart walls [23,24,25,26,27,28]. In addition, it is characterized by high variability in time due to fetal and maternal movements [3,29,30] and changing of transducer position in relation to a moving signal source—the fetal heart [31,32,33]. Identification of the heartbeat occurrence, based on a simple peak detection in looking for characteristic component, is very inaccurate due to its variable shape [23,34]. In addition, any interfering impulses of sufficiently high amplitude can be identified as successive beats [23,35,36,37]. All of these makes practically impossible to indicate points equivalent to the R-waves in FECG [38]. Therefore, for FHR determination based on the US signal, correlation methods considering the full impulse shape are used [33,35,39,40,41,42].

The dominant method is the autocorrelation function (AF), which comparing to the cross correlation, is easier to perform and does not require selecting a heartbeat pattern that makes it resistant to signal interferences [43,44,45]. Unfortunately, only the periodicity is estimated, not the exact locations of subsequent heartbeats in the analyzed signal. Since measurement of the T_i_ is repeated in windows covering at least two complete cardiac cycles, a single heart interval can be represented by more than one measurement. Such signal form makes it impossible to reliably assess a beat-to-beat variability. The physiological FHR range is from 60 to 240 bpm (which corresponds to T_i_ intervals from 1000 to 250 ms). Therefore, not to miss any cardiac cycle, subsequent FHR values must be determined at least every 250 ms. Such repetition time is a widely accepted standard at which Doppler ultrasound monitor provides a new FHR value at its output.

To date, the possibilities of parameterization and objectification of the FHR variability evaluation relate to accurate quantitative signal analysis carried out by dedicated computer-aided fetal monitoring systems [46,47,48,49,50,51]. Automated signal analysis ensures repeatability of interpretation [52,53,54,55,56], as well as the extraction of information hidden for visual assessment, such as instantaneous FHR variability at a beat-to-beat level [57]. At turn of the 60 s and 70 s, several different indices were defined for quantitative description of the instantaneous FHR variability [58]. Most of these definitions assume that the analyzed signal comes from an invasive direct FECG, i.e., the indices are calculated based on T_i_ intervals determined between the R waves. In other words, the analyzed FHR signal has a form of time series of events, in which each cardiac cycle is represented by a single measurement only. Nevertheless, a direct application of the definitions originating from FHR acquired via FECG, to the FHR determined from Doppler US signal, does not give good results. Especially the short-term variability indices, calculated from measurements occurring every 250 ms, show significant errors [59,60,61,62].

Therefore, attempts have been made to process the FHR signal from a time series of evenly spaced measurements into a time series of events representing subsequent heartbeats [63,64,65]. Despite the improved method for determination of FHR variability indices [65], their accuracy was still limited due to a wide autocorrelation window resulting in averaging of several neighboring intervals. The window width selection is a compromise between noise immunity and measurement accuracy. In practice, the AF windows of a few seconds are used, which ensures the continuity of periodicity measurement even in the presence of interferences from fetal movements, however at a cost of the measurement accuracy. The determined interval value does not then represent a single cardiac cycle, but an averaged value of few last beats [36,62,64,66]. It does not affect the results of classical FHR analysis—the baseline estimation or detecting accelerations and decelerations, because such analysis is carried out on a signal averaged in 2.5 s periods [62]. However, averaging of measurements practically prevents a reliable STV determination regarding beat-to-beat changes [62,65].

It seems that analysis of the US signal inside a fetal monitor do not use its full potential. This results partly from a lack of requirements for this type instrumentation, due to relatively short history of fetal monitoring systems enabling automated FHR analysis with beat-to-beat accuracy. The question arises whether, by improving the built-in algorithm for determining the periodicity in a raw Doppler ultrasound signal, it is possible to obtain measurements close to reference values from the FECG. Peters [66] developed an algorithm for determining the FHR from US signal, providing it in a form of time sequence of events, which enabled correct determination of indices describing the FHR instantaneous variability [66]. The sequence of events was obtained by using markers that approximately corresponded to subsequent heartbeats. They were used to select the signal fragments for which the AF was determined. Peters showed a high correlation with the FHR signal determined from direct FECG. However, he examined only one short signal and did not evaluate an interval measurement accuracy.

Such evaluation was made in [67], where an autocorrelation window comprising two cardiac cycles was positioned in US signal based on the simultaneously recorded FECG. The research concerned different values of window shift in relation to its initial position, determined by an occurrence of the R wave. Unfortunately, the work is only theoretical, because the method, as requiring an additional signal source, is not suitable for any practical use in fetal monitor.

Al-Angari has also investigated the problem of determining the FHR signal from the Doppler ultrasound, as time series of events [68]. However, the obtained FHR signals were characterized by unusually high variability. Moreover, the source of reference FHR was a FECG signal, but recorded indirectly from a maternal abdomen. Jezewski also dealt with the analysis of a raw US signal [36] recorded together with a reference direct FECG signal. The width and step of the AF window were adapted to the previously determined interval value. The efficiency of a new method was estimated by the interval measurement and de Haan’s variability index determination errors [58]. Thus, the obtained results confirmed the high efficiency, but only in relation to a single FHR variability index [64].

The cited works do not allow us to answer the previously asked question about the possibility of determining the FHR from a raw US signal with the accuracy similar to the reference direct FECG. Therefore, the authors of this work conducted comprehensive research on the Doppler ultrasound signal describing the mechanical activity of a fetal heart. The aim of this research was to develop new methods that will both improve the accuracy of FHR determination and provide the resulting signal in form of time series of events. Achieving of these two goals should result in a more accurate description of instantaneous FHR variability, and thus a more effective early detection of fetal distress.

The goal of our research was to develop a complete channel for processing the Doppler ultrasound signal enabling precise periodicity measurement. First, the information content of the US signal was analyzed-two subranges of Doppler bandwidths were separated using bandpass filtering to describe slower heart wall movements and valve motions of a higher speed. In a next stage, the US envelope was determined, and a classic low-pass filtering and Hilbert transform were considered. To determine the FHR signal, the autocorrelation function supported by the trapezoidal prediction function was used. In final stage, we proposed two different methods providing the FHR signal in a form of time series of events: first using correction of duplicate measurements and second based on segmentation of instantaneous periodicity measurements. In addition, very important was the original methodology for evaluating efficiency of the proposed algorithms, which was based on automated synchronization of examined FHR signals. All these aspects are important because there are no other publications comprehensively evaluating the FHR signal determination accuracy, both by estimating cardiac interval Ti accuracy and calculating clinically crucial indices describing the instantaneous FHR variability and additionally using direct fetal electrocardiography as reference data.

## 2. Methodology

Using an ultrasound method, determination of a fetal heart rate is based on analyzing the Doppler effect, i.e., a frequency change of US beam reflected from moving parts of a fetal heart. The received returning echo is a signal with a frequency of the US wave modulated by the speed of a given moving structure. After amplification it is demodulated for separation of particular Doppler components. Then the signal is converted into a digital form, and a further processing is carried out—leading to the FHR determination.

**Figure 1:**
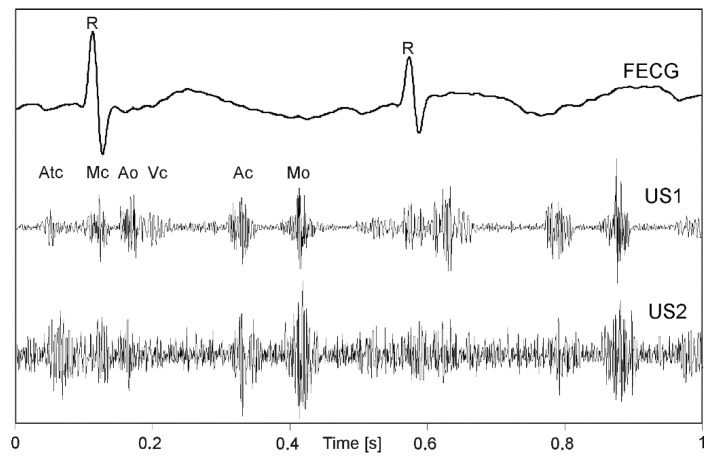
An example of the one-second direct electrocardiogram (FECG) with Doppler ultrasound signals from two transducers (US1, US2) attached to maternal abdomen in different position relative to a fetal heart. In the US signal the cardiac phases are observed: opening and closing the crescent—Ao and Ac, atrioventricular valve—Mo and Mc, atrial wall contraction—Atc and walls heart chambers—Vc.

When all phases of a cardiac cycle, originating both from valve and wall movements are present (Figure 1) much more complex signal structure (US1, US2) in relation to a simultaneously recorded FECG can be noticed. The amplitude and shape of individual components change during long-term monitoring, as the object (fetus) can change its position relative to a transducer [31]. The FECG additionally presented in Figure 1, allows for detailed identification of cardiac phases observed in signals of mechanical heart activity.

**Figure 2:**
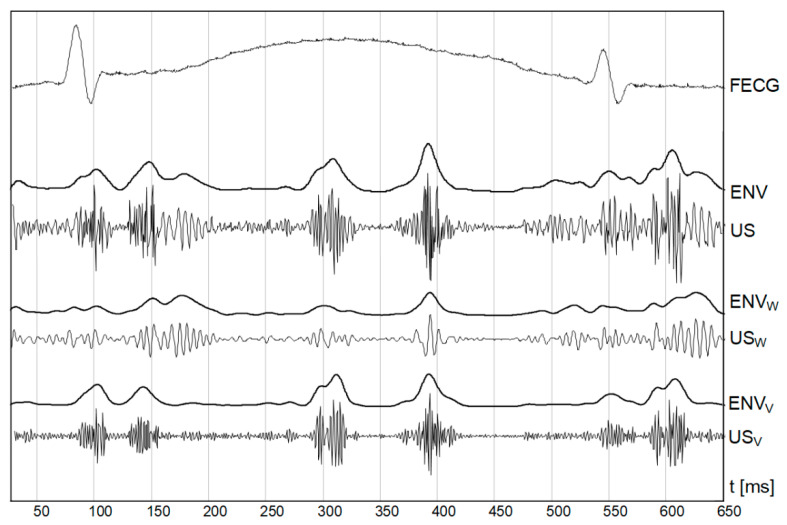
Doppler ultrasound signal (US) with separated components relating to the fetal heart wall (US_W_) and valve (US_V_) movements. Corresponding envelopes (ENV_W_ and ENV_V_) are shown together with an envelope of full signal (ENV).

In addition to an occurrence rate, particular cardiac phases are characterized by different bandwidths. The lower frequencies correspond to the heart wall, while the valve movements are represented by higher frequency components (Figure 2). The US signal envelope describing the valves is characterized by higher dynamics, and narrow peaks enabling accurate cardiac cycle length estimation. In turn, the envelope signal from the walls is much more smoothed, with wider peaks which reduces periodicity measurements accuracy. Additionally, a risk of erroneously doubling the heart rate increases, since the signal envelope fragments during the systole and diastole phases are very similar.

### 2.1. Data Acquisition

The research material used for this study was collected at the Obstetric Clinic of the Medical University of Silesia in Zabrze. It was carried out by investigators from the Medical University of Silesia during development the project “Application of direct electrocardiography for intrapartum assessment of fetal distress “(Bioethics Committee approval number: NN-013-345/02)”.

The fetal monitor used in this study enables to acquire a raw Doppler US signal (1 MHz) obtained after the demodulation process. The FHR_CTG measurements calculated by built-in fetal monitor software were accessible on a digital output.

Since the additional direct FECG recording (for determining FHR_E) required measuring electrode placed on fetal head, all signals were recorded as part of a routine procedure for labor monitoring. Six sessions (39 ÷ 41 week of pregnancy) lasting from 280 to 1263 s were carried out. The final research material included 58 min of simultaneously acquired: Doppler US signals being the input data for the algorithms investigated, original FHR_CTG signals determined by a fetal monitor, as well as reference information in the conducted research—FHR_E signals. The resolution of both FHR_CTG and FHR_E signals were 0.25 bpm.

### 2.2. Signal Processing

The goal of the research was to develop a complete channel for processing the Doppler ultrasound signal enabling precise periodicity measurement. The basic assumption was that the resulting FHR signal should be in form of time series of events, i.e., each subsequent interval between two heartbeats should be represented only by a single measurement—like in FECG.

**Figure 3:**
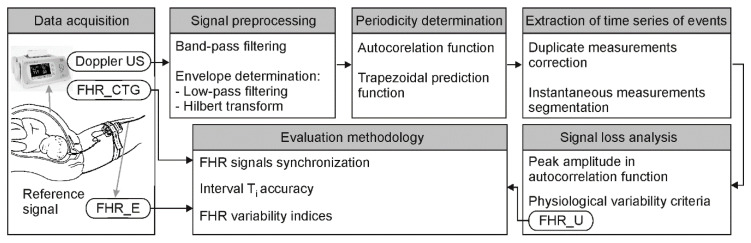
Block diagram generally describing the procedure of subsequent signal processing stages and methods for final evaluation of the results obtained.

The first stage of the research focused on optimal representation of the Doppler ultrasound signal (Figure 3). The signal information content was analyzed for the influence of individual cardiac phases on the accuracy of measurement. Two bandwidths of Doppler frequencies were separated using bandpass filtering: one describing wall and one relating to valve movements. The next stage concerned reduction of signal complexity by calculating its envelope. Classic low-pass filtering and the Hilbert transform were considered. For methods evaluation, the FHR values were determined by AF, calculated every 250 ms.

During the next stage, we focused on tuning algorithms for determining the FHR signal: selection of autocorrelation windows parameters both for the autocorrelation and prediction function. Next, two different methods were developed to provide FHR signal in form of time series of events: using correction of duplicate measurements [65] and basing on segmentation of instantaneous periodicity measurements. During the algorithm development, different factors were recognized as having a major impact on the final Ti measurements. No less important was the final measurements validation, if the determined interval value was correct, due to interferences originating from maternal heart activity or fetal movements.

The final stage of the study concerned metrological assessment of the proposed methods in relation to reference values provided by a fetal electrocardiogram. To validate the results, a precise synchronization of FHR signals was very important to ensure comparing the corresponding intervals. Two different procedures were proposed to estimate the inconsistency. A direct comparison of signals by the differences of instantaneous FHR values was accompanied by signal loss analysis and evaluating the signals impact on the indices describing long-term and short-term FHR variability.

#### 2.2.1. Signal Preprocessing

**Figure 4:**
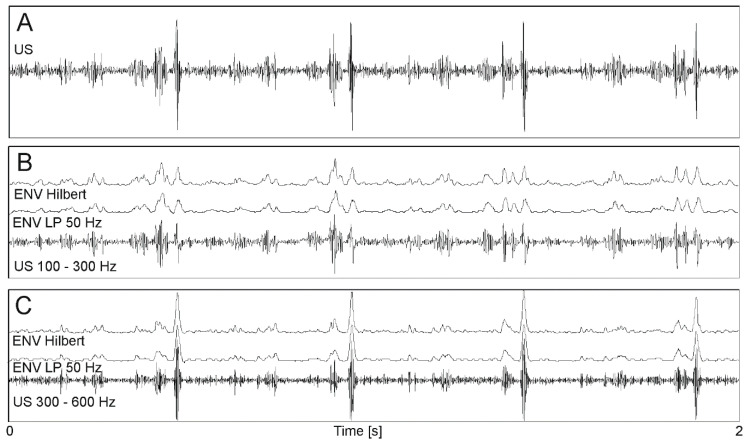
Doppler ultrasound signal processing. (**A**) original signal after demodulation; (**B**) signal after band-pass filtering 100 ÷ 300 Hz (wall movements) and two envelopes determined using 50 Hz low-pass filter and the Hilbert transform; (**C**) signal after band-pass filtering 300 ÷ 600 Hz (valve movements) and two envelopes as in B.

In the first step, the appropriate signal bandwidth was selected to obtain “optimal” signal information content for more accurate interval measurement. Initially, it was assumed that the entire bandwidth, corresponding to both heart valve and wall movements, should be analyzed. It is difficult to measure signal periodicity precisely because each cardiac cycle in the envelope signal is represented by several impulses related to different cardiac phases. The number of events observed in a signal can be reduced by separation of signal components corresponding to different motion speeds of heart structures. In the case of fetal monitoring, it is assumed that using an ultrasound transducer emitting with 1 MHz, wall and valves movements are within bandwidth of 100 ÷ 600 Hz [36] Using band-pass filtering the subranges were separated, covering frequencies from 100 to 300 Hz (wall movements—Figure 4B) and from 300 to 600 Hz (valve movements—Figure 4C). Filtering below 100 Hz enabled to remove components originating mainly from fetal trunk and limbs movements, while above 600 Hz from flows in maternal blood vessels.

The next step involved determining the Doppler US signal envelope aimed to reduce the signal complexity. The easiest way to obtain an envelope is to convert a bipolar signal into a unipolar one using a module function and next a low-pass filtering. Too low filter cutoff frequency causes blurring of phases observed in a cardiac cycle, which, leads to lowering the AF peak and reducing measurement accuracy. On the other hand, too high cutoff frequency (e.g., 150 Hz) incorrectly shifts the peak due to high AF corrugation. The filter parameters should be adjusted to Doppler frequencies, which are proportional to the 1 MHz. During the tests, the envelope signal was determined using different cutoff frequencies (25, 50, 75, 100, 150 Hz), as well as without any additional filtering.

The Hilbert transform was used as an alternative method for determining the envelope [33]. The envelope determined using the Hilbert transform was characterized by quite high variability, especially in fragments of high dynamics. Therefore, a moving average filter (MA) was additionally used to smooth the envelope. The best smoothing while maintaining the impulse shape, is ensured by averaging over 11 or 21 signal samples (established empirically). For further studies, the envelope was determined using the Hilbert transform without additional filtering and MA with a window of 11 or 21 samples. Figure 4B,C shows exemplary envelopes determined with a low-pass filter with a cutoff frequency of 50 Hz and using the Hilbert transform.

To assess the impact of the bandwidth applied on the accuracy of measurements at this stage of study, a simple AF determined every 250 ms within windows of 1.5 s was used. Because at this stage of analysis we just want to compare two methods for envelope determining, so such approach, which is a compromise between ensuring the continuity of measurements and their accuracy, is the most right. Additionally, the AF threshold relating to low level of signal quality and thus defining the FHR signal loss was empirically determined. For this purpose, the most disturbed signal fragments were analyzed, in which in the absence of fetal heart episodes, many local AF peaks were observed, whose amplitude usually did not exceed 0.1. Therefore, if the amplitude of AF peak did not exceed P_TH_ = 0.1, a loss marker was set in the resulting FHR signal. Such AF threshold is more rigid than 0.15 proposed in the study [36].

#### 2.2.2. Periodicity Determination

Two different methods for determining the autocorrelation function were used. The first is the detection of the AF peak calculated arbitrarily every 250 ms. In the second case, AF is calculated more frequently with every 25 ms, and the outlier values most often caused by temporary interferences, are rejected from a group of measurements. An important factor affecting both the measurement accuracy of T_i_ interval value and the level of FHR signal loss is the AF window width. It is required that the window should cover at least two of the same cardiac phases belonging to two consecutive cardiac cycles. The multiphase motion process observed in US signal means that determination of the T_i_ interval does not require a window including two full cardiac cycles. Even if certain cardiac phase will occur only once in a narrower window, then most likely the other phase will be represented twice, allowing correct measurement. A narrow AF window means minimization of the averaging effect over several cycles, and thus a higher measurement accuracy. However, too narrow of a window may prevent correct measurement, which will result in FHR signal loss. Jezewski [36] set the minimum window width at 1.5 of the last measured interval duration. The advantage of this adaptive window width selection is reduction of necessary calculations, especially in case of short T_i_ intervals, which may be important in practical implementation in battery-powered mobile fetal monitors. On the other hand, so strong dependence of the calculation process on previously measured values can cause a permanent switch to an incorrect range of T_i_ values.

**Figure 5:**
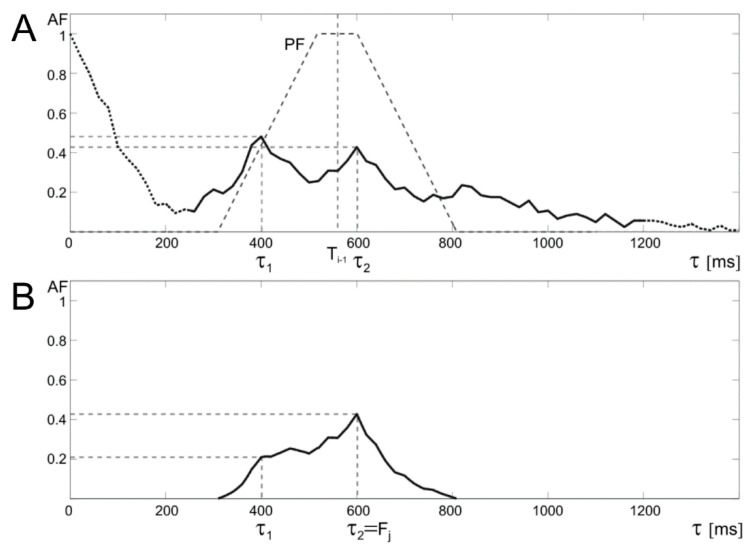
Idea of a trapezoidal window applied for the prediction function prediction function (PF). The upper graph (**A**) shows the autocorrelation function with PF, whose center is determined by the last correctly determined value of the T_i−1_ interval. The lower graph (**B**) shows that correction of autocorrelation function (AF) by trapezoidal PF which enables determination of the correct periodicity value (τ2) and thus the instantaneous measurement F_j_, despite the distortions in the US signal, which gives false AF maximum (τ1) before PF application.

The algorithm for the periodicity determination assumes that when the signal quality is satisfactorily high, the maximum of AF found is likely to correspond to the T_i_ interval value in US signal. To prevent erroneous measurements for low-quality signal fragments (AF peak amplitude < 0.5), the prediction function (PF) was proposed, where recently determined interval value is taken as an expected value for the next one. Such AF peak amplitude used in prediction function was determined empirically based on analysis of problematic cases observed in available signals. In the absence of interferences, the most frequently observed AF peak amplitudes were usually above 0.6. In the presence of additional disturbing episodes observed due to fetal movement activity, the AF peak amplitude was just below 0.5. The value of the AF is corrected by using a trapezoidal PF window, with a selected width of the lower base, a base ratio of 1:4 and the center located at the point τ = T_i−1_ (Figure 5). The proposed solution is a modification of the approach from [36], where a triangular window was used, which however exposed too much the last measured value. In cases of increased heart rate changes, e.g., on acceleration and deceleration slopes, where the differences of successive intervals are significant, the use of a triangular window caused erroneous measurements.

The new trapezoidal window causes that the AF amplitude for shifts τ close to the values of recently determined T_i_ remains unchanged. While for less likely intervals (τ values significantly different from T_i−1_) the amplitude is reduced according to the applied PF. In addition, it was assumed that if the current interval was determined using a prediction algorithm, then it does not modify the PF position for the next iteration. Finally, the T_i_ takes the value of τ for which the AF(τ) reaches maximum value.

In the next part of investigation, we developed two independent methods based on the autocorrelation function, which allow the resulting FHR signal to get the required form of time series of events. For this purpose, a method of correcting duplicate measurements [65] was used, as well as a segmentation of instantaneous periodicity measurements.

#### 2.2.3. Extraction of Time Series of Events

**Figure 6:**
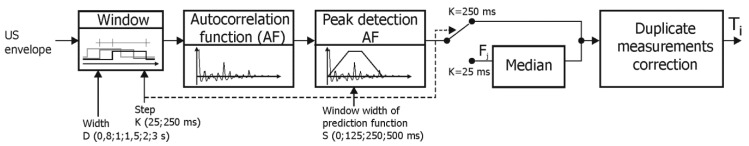
General block diagram of the D1 algorithm for determining the instantaneous value of fetal heart rate (T_i_ interval) based on a raw Doppler US signal.

The first method (D1) for determining the fetal heart rate signal from the Doppler US signal provides the FHR value every 250 ms (like in classical monitors), after which a correction of duplicate measurements proposed in [65] is applied (Figure 6). The AF was calculated every 250 ms, but sometimes also more often (every 25 ms) and then the representative FHR value was the median of 10 consecutive instantaneous measurements F_j_. According to [36], the minimum window width was set at 800 ms, which is 1.5 of maximum interval value (533 ms) in the reference signal, and other tested widths were: 1, 1.5, 2 and 3 s. The impact of different PF window widths (S) on the final measurements was also tested with 125, 250 and 500 ms, which were adopted basing on visual analysis of the AF. Value S = 0 means that PF was not used, and then the position of maximum peak of AF (in the physiologically acceptable range from 30 to 2000 ms) defined periodicity.

The FHR values determined every 250 ms, underwent correction of duplicate measurements to obtain a signal in the form of series of events according to the authors’ algorithm [65]. The aim of the algorithm for identifying and correcting duplicate measurements is determination of the number of true heart cycles represented by a sequence of instantaneous measurements. For each analyzed sequence, the Min and Max values are determined, defining two different numbers of such intervals. If the rounded Min and Max values are equal, then this value represents the only correct solution. In the case of different values, determination of the number of real heart intervals is based on analysis of time dependencies as well as assessment of probability of a given solution existence.

**Figure 7:**
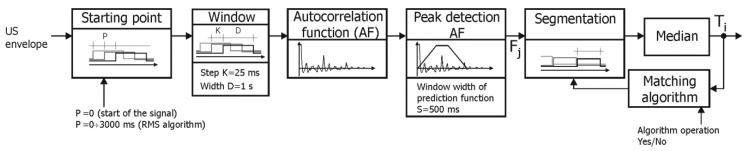
General block diagram of the D2 algorithm for determining the fetal heart rate based on a raw US signal.

In the D2 method, the autocorrelation function is determined in a window shifted with 25 ms (Figure 7). Subsequent values of instantaneous periodicity F_j_ are determined using the prediction function (as in the D1 method). The next stage is segmentation of measurements F_j_, which identifies a group of subsequent measurements, which will then be used to calculate the resulting interval T_i_. Segmentation relies on continuous computation of a median value until a time being the product of number of measurements and repetition period (25 ms) exceeds the current median value. Then the median becomes the searched T_i_ value and the procedure is repeated. Unfortunately, the obtained results depend on a starting point determining the phase shift between segments and series of F_j_. Two approaches are proposed to reduce this unwanted effect.

**Figure 8:**
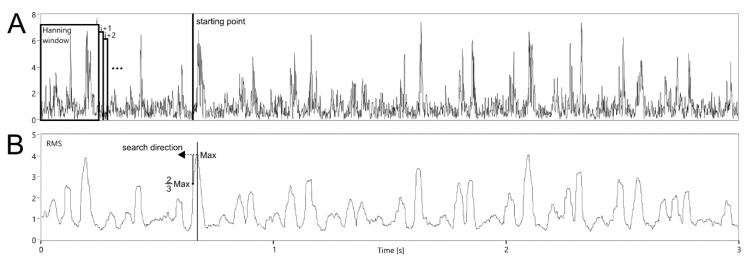
Idea of searching a starting point within the analysis of the envelope signal (**A**) for segmentation of instantaneous F_j_ values using the D2 algorithm based on RMS function (**B**).

The first one is to specify a starting point other than the signal beginning (*P* ≠ 0). If the point correctly represents a cardiac cycle beginning, then the value of T_i_ will be determined only on the F_j_ corresponding to the considered interval. Otherwise, some of them will relate to the neighboring T_i−1_ interval, causing erroneous value of the current T_i_. To find the starting point, RMS value was calculated using the envelope signal. Various windows (100, 200, 250, 500 and 1000 ms) moved with 1 ms step were tested (Figure 8). This range of RMS window width was adopted from observation of information content in terms of identifying the patterns representing fetal cardiac phases. For the first three seconds of the signal, the maximum value of the RMS function was determined. Then, going back to the beginning, the starting point P was set as soon as the current RMS value dropped below 2/3 of the maximum.

**Figure 9:**
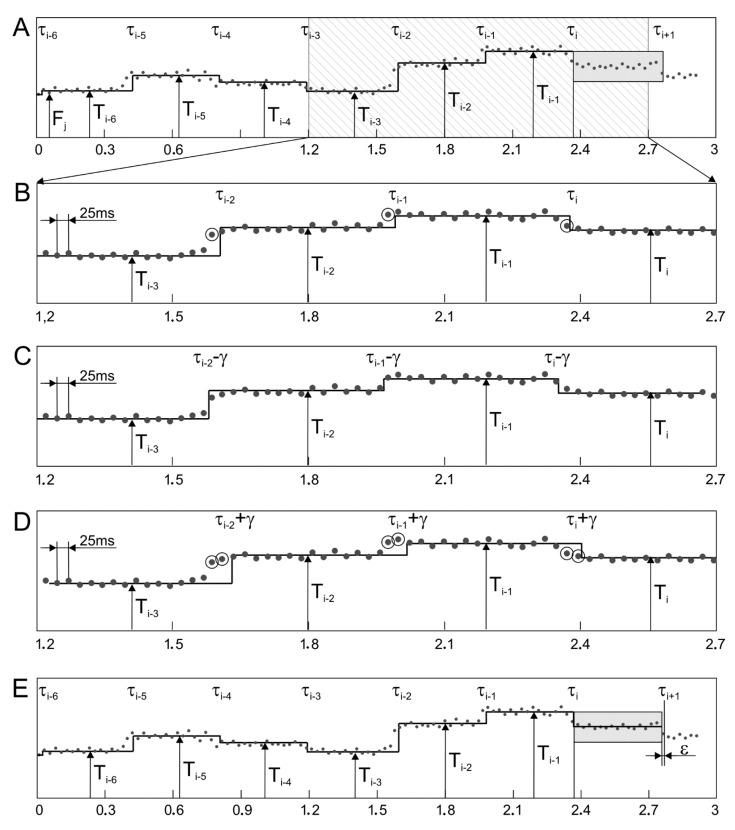
Operation of matching algorithm in the segmentation method based on values of instantaneous periodicity measurements F_j_ (**A**). Three middle graphs (**B**–**D**) show the principle of determining the correction parameter. For seven most recently determined intervals, matching to instantaneous measurement values is tested. If the average difference between F_j_ values and the corresponding values of the intervals T_i_ decreases when the intervals are shifted by γ or −γ (repetition step for measurements F_j_), then the length of the determined interval is corrected by ε = γ/5 or −ε = γ/5, respectively (**E**).

The second matching algorithm (MAA) concerns the correction of already determined T_i_ interval value and compensates the effect of shift between the determined segments and the F_j_ series (Figure 9). The matching is controlled by a mean value of the absolute differences calculated between particular F_j_ and mean value of all F_j_ measurements within the analyzed segment. It makes that the value is calculated for three different cases: no shift of time stamps of the analyzed segments, a negative shift and a positive shift. As many as seven recently determined segments are analyzed, which ensures that none of distorting factors affect the final result of matching. If the minimum difference corresponds to a nonzero shift value, then an appropriate phase correction is applied to the time stamp of the interval beginning, while the T_i_ value itself remains unchanged. The details of the algorithm operation are presented in Figure 9. In the first stage, the value of the T_i_ interval is calculated as the median among measurements of instantaneous periodicity in the range between τ_i_ and τ_i_ + T_i−1_ (Figure 9A). Then a mean difference is calculated between F_j_ values, obtained for seven intervals from τ_i−6_ to τ_i_ + T_i−1_ and for values from T_i−6_ to T_i_, representing these intervals (Figure 9B). To emphasize the time dependencies, only a signal fragment with a length of 1.5 s is presented and the measurements F_j_ (which differ significantly from a given T_i_ interval), are marked with a circle. In the next stage, the calculations are repeated for time markers τ_i−6_ ÷ τ_i_ + T_i−1_ shifted by γ = ±25 ms, i.e., by a period between two consecutive fetal heart rate measurements F_j_ (Figure 9C,D). If the minimum average difference is in the time stamp shift, the correction coefficient ε takes a value of ±1/5·γ, i.e., ±5 ms, otherwise it is 0 (Figure 9E). Then a time stamp value of a beginning of the next interval is determined as τ_i+1_ = τ_i_ + T_i−1_ − ε, the increment of the variable “i” and the repetition of calculations for subsequent intervals are carried out.

A similar correction method was used in [36], however, only the last three T_i_ intervals were analyzed, which was not always enough. If some of F_j_ measurements represented incorrect values, proper matching was difficult or even impossible. A similar matching problem concerned the signal fragments with very low variability—several successive intervals had very similar values. Since in [36] AF was calculated with variable step (depending on recently determined T_i_), the value of the correction was also variable and equal to 0.25 of the currently adopted step for calculating F_j_.

#### 2.2.4. Signal Loss Analysis

Additional interferences, originating from fetal movements or maternal blood vessels, may result in shortening of the determined cardiac cycle length or its lengthening if one of heartbeats is missed. To ensure a high accuracy in measuring intervals, an elimination of significant errors is required. Since it cannot be done as part of the AF, a preliminary acceptance criterion should be applied to T_i_ intervals already at the stage of AF calculation. The AF amplitude is an indicator of a signal quality and consequently, the correctness of the T_i_ calculation. Instantaneous periodicity measurements for which the AF peak is lower than the threshold P_TH_ = 0.1 are omitted. The threshold value was determined empirically, as it was described Signal preprocessing subsection. Intervals considered to be invalid cause that signal loss markers are inserted in the FHR.

The next stage concerns the FHR limits resulting from physiology. The classic van Geijn criterion is used to identify and remove artifacts [69]. However, the results of our research indicate that in some situations the criterion does not work correctly. Too wide range of T_i_ instantaneous changes is considered acceptable, mainly to ensure correct interpretation of values within the slopes of deceleration and acceleration patterns. Unfortunately, very often these values are incorrect and distort the results of beat-to-beat variability analysis. Therefore, more rigorous validation criteria are proposed, where the T_i_ interval must meet the condition:

(2) $$T_{i-1}-0.1\cdot \Delta_{i-1}<T_{i}<T_{i-1}+0.15\cdot \Delta_{i-1}$$

where:

(3) $$\Delta_{i-1}=\{\begin{array}{cc} T_{i-1}-300\;\mathrm{ms} & {\mathrm{dla}\;T}_{i-1}\geq 320\;\mathrm{ms}\\ 20\;\mathrm{ms} & {\mathrm{dla}\;T}_{i-1}<320\;\mathrm{ms} \end{array}$$

The T_i_ interval is accepted if it belongs to a group of at least three successive intervals meeting the above condition. In some cases, not only artifacts are removed from the signal, but also the correct interval values, especially within the slope of FHR acceleration or deceleration patterns. To prevent this, validation takes place in two directions of the timeline, accompanied by a change of thresholds. Only the T_i_, which does not meet the criterion for both directions, is considered as incorrect. The final verification of T_i_ values initially classified as incorrect is carried out based on the analysis of the monotonicity of beat-to-beat changes. A given T_i_ interval is considered incorrect, if the differences between itself and neighboring intervals are of the same sign and the product of these differences is greater than 35 ms^2^, which was determined on the basis of research presented in [70].

### 2.3. Evaluation Methodology

To assess the differences between FHR signals obtained by the investigated methods in relation to the reference values obtained from a direct fetal electrocardiography, a set of descriptive statistics parameters calculated for all 58 signals was determined:

$\bar{\Delta T_{i}}$ —mean value of T_i_ interval differences $\Delta T_{i}$ between the investigated and reference method;
$\mathrm{SD}\_\Delta T_{i}$ —standard deviation of the differences $\Delta T_{i}$;
$\left|{\Delta T}_{i}\right|$ —measurement error of the i–th interval, as an absolute value of $\Delta T_{i}$;
$\bar{\left|{\Delta T}_{i}\right|}$ —mean value of the measurement error $\left|{\Delta T}_{i}\right|$.

Each of the FHR determination methods introduced a variable shift of the calculated intervals in relation to the source Doppler US signal. It is caused by the fact that different window width and different starting point (depending on a signal content) were used for AF. In addition, reference signals were obtained from a direct FECG, hence the presence of an additional time shift. Therefore, to ensure the reliability of the analysis, it was very important to synchronize the FHR signals to make certain the comparison of corresponding cardiac cycles in the investigated signal (T_i__B) and the reference one (T_i__E). Due to the size of the research material, synchronization was carried out automatically.

**Figure 10:**
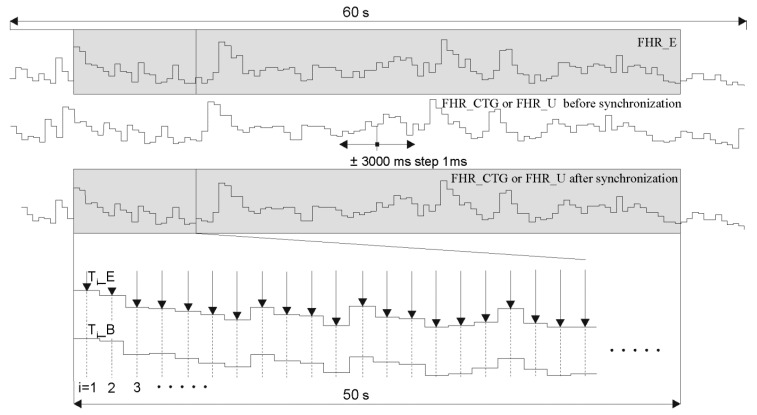
Synchronization procedure of the fetal heart rate signals: FHR_E—obtained from the reference direct FECG, FHR_U—determined by one of the methods based on a raw Doppler US signal and FHR_CTG—acquired directly from a fetal monitor output.

In the first step, both FHR signals were presented in a form of time series (Figure 10). Then the FHR_U signal determined by the investigated method was shifted in the range +/– 3000 ms, in steps of 1 ms. The mean interval measurement error was calculated for each shift. It was assumed that subsequent values $\left|{\Delta T}_{i}\right|$ are calculated the middle of the duration of subsequent reference intervals (indicated by arrows in Figure 8):

(4) $$\left|{\Delta T}_{i}\right|=|T_{i}\_B-T_{i}\_E|$$

where: T_i__B—value of the i-th interval determined with the investigated method T_i__E—reference value of this interval obtained from a FECG.

In addition, only intervals within the range from 5 to 55 s (of a 60-s reference signal) were considered. It was found that the correct synchronization refers to the shift value for which the minimal mean $\bar{\left|{\Delta T}_{i}\right|}$ error is obtained.

A very important FHR quality measure is the signal loss level, which has a major impact on the results of automated analysis [32,71]. Very high measurement accuracy may be a result of rejecting too large number of “uncertain” measurements during calculations. Only the evaluation of the T_i_ measurement error together with the amount of FHR signal loss enable a reliable performance assessment of a given method. FHR signal loss is calculated using loss markers determined during a method verification. As in the case of the T_i_ error, in order to standardize the results, an analyzed fragment concerned a time interval from 5 to 55 s.

Considering diagnostic significance of indices for quantitative description of the instantaneous FHR variability (both short- and long-term), the method evaluation was not limited only to descriptive statistics of the measurement error. Detailed error analysis in variability indices determination was considered as very important criterion for assessing the efficiency of the investigated method. For purposes of the study, six short-term (named with S followed by an index author’s name) and six long-term (named similarly with L) FHR variability indices [58] were selected. They were calculated over the corresponding fragments from 5 to 55 s for the FHR_CTG and FHR_U signals and compared with the corresponding reference values. Relative errors of indices determination were calculated, for example, the determination error for the S_DAW index is described by a formula:

(5) $$\delta S\_\mathrm{DAW}\left[\%\right]=\frac{S\_\mathrm{DAW}-S\_{\mathrm{DAW}}_{E}}{S\_{\mathrm{DAW}}_{E}}\cdot 100\%$$

where:

S_DAW—the index value provided by an investigated method;
S_DAW_E_—the reference value calculated from FECG.

## 3. Results

### 3.1. Study of Sensitivity

In the first stage, based on interval measurement error and FHR signal loss (Table 1), the influence of the Doppler bandwidth and the method for envelope signal determination was evaluated. The highest errors $\bar{\left|{\Delta T}_{i}\right|}$ relate to the band associated with heart wall movements (from 100 to 300 Hz) and the main reason is the more blurred impulse shape in the envelope, which leads to less evident peak in AF. Hence, interfering impulses may cause slight shifts in the position of the dominant peak. A much lower T_i_ error was noticed for the higher (from 300 to 600 Hz, corresponding to heart valve movements) and full (from 100 to 600 Hz) bandwidth. This means that the strongest impact on a measurement accuracy have valve movements, represented by very sharp peaks in the envelope and AF.

Limiting the signal bandwidth from 300 to 600 Hz did not lead to a clear improvement in measurement accuracy and a higher signal loss level was observed than in the case of a full bandwidth. Analyzing the results from Table 1, we decided to select for further experiments four combinations: 300 ÷ 600 Hz bandwidth and 50 Hz filter, 300 ÷ 600 Hz bandwidth and Hilbert filter with additional moving average over 21 samples (MA21), 100 ÷ 600 Hz bandwidth and 100 Hz filter and finally 100 ÷ 600 Hz bandwidth and Hilbert filter with MA21. The results for these combinations are in bold in Table 1.

#### Extraction of Time Series of Events

The D1 method for determining the FHR from the Doppler US signal relies on cyclical calculation of subsequent FHR values using the autocorrelation function, followed by the correction of duplicate measurements [65]. In the first approach, the AF was determined with a repetition cycle (step) K = 250 ms, and the measurement result was the final instantaneous FHR value. In the second approach, the periodicity during each cardiac cycle was measured more frequently (K = 25 ms), and the final value was determined as a median of 10 consecutive measurements. Additionally, the usefulness of the periodicity prediction function was tested using different window widths S. The obtained results are presented in Table 2.

It can be seen that repetition cycle K had no significant impact on the results. Both the errors and signal loss calculated for a specific combination of the PF window and the envelope determination method, have a similar value. In turn, when analyzing an effect of the prediction function, the smallest T_i_ measurement error was obtained with no PF. Regardless of the envelope determination method, all errors were in a narrow range from 1.89 to 1.96 ms. Using a narrow PF window (S = 125 ms), both measurement errors and signal loss were much higher. Increasing the width S significantly improved the results—the values of $\bar{\left|{\Delta T}_{i}\right|}$ were like those obtained without using the prediction function, while the signal loss was several times lower. Generally, the best results were obtained using PF with S = 500 ms and 300 ÷ 600 Hz bandwidth, hence further experiments were carried out with these settings.

Our study of sensitivity regarding autocorrelation function determination and T_i_ interval measurement was completed with optimization of the AF window width D. The analysis covered five different widths: 0.8; 1; 1.5; 2 and 3 s, and results obtained for various combinations of control parameters are presented in Table 3. The calculations were carried out for the repetition cycle K equal to 25 and 250 ms, bandwidth of 300 ÷ 600 Hz corresponding to heart valve movements, two types of envelopes and with or without PF. It can be seen that using too short AF window (0.8 s) resulted in very large FHR signal loss. In the case of limited number of phases visible in a cardiac cycle, a short AF window did not always comprise two phases of the same type, thus preventing periodicity measurement. With an increase of D, this problem becomes less significant, and moreover, the level of FHR signal loss decreases because short interferences have less influence on the AF shape. Signal loss is strongly dependent on the prediction function (for each AF window width), the use of PF with S = 500 ms reduces the signal loss several times.

Neither the prediction function nor the envelope determination method affects the $\bar{\left|{\Delta T}_{i}\right|}$ values, which depend only on the AF window width D. Definitely the lowest errors refer to the one-second width (from 1.2 to 1.26 ms). For a narrower window, some of the erroneous measurements, but fulfilling van Geijn criteria, are considered correct, causing a slight increase in the error value. In turn, a wider window means that the resulting measurement represents a greater number of consecutive cardiac cycles, which leads to averaging of T_i_ values and to error increase. Ultimately, the best results both for $\bar{\left|{\Delta T}_{i}\right|}$ and signal loss, were obtained for AF window of 1 s with repetition of measurements every 25 ms and using an additional prediction function.

Finally, the T_i_ interval determination using two different signal envelopes (low-pass filtering with a 50 Hz cutoff and the Hilbert transform), was used in the algorithms to extract the FHR signal in the form of time series of events. After correction of duplicate measurements [65], for envelope with low-pass filtering, the error $\bar{\left|{\Delta T}_{i}\right|}$ increased from 1.24 ms for K = 25 ms (Table 3), to 1.98 ms for the representation as time series of events. For the Hilbert transform, the error values were 1.25 ms and 1.91 ms after the correction algorithm (Table 3).

As an input signal for D2, we used two optimal (selected within D1) signal envelopes determined by 50 Hz low-pass filtering and the Hilbert transform. The AF was calculated every 25 ms in one-second window, whereas PF with 500 ms window was used to determine the T_i_ value.

During the studies it was tested how the obtained results depend on the starting point selection, i.e., the location of the first AF window in the envelope signal. Table 4 presents the $\bar{\left|{\Delta T}_{i}\right|}$ and FHR_U signal loss for different starting points: arbitrarily selected at the signal beginning, as well as five other points determined using the RMS method calculated in windows: 100, 200, 250, 500 and 1000 ms. In addition, we estimated usability of the developed matching algorithm (MAA) with continuous correction of the determined sequence of events in relation to the series of instantaneous measurements F_j_.

The starting point selection practically did not affect the accuracy of the T_i_ measurement. Without additional matching algorithm, regardless of the envelope type, the mean error $\bar{\left|{\Delta T}_{i}\right|}$ ranged from 1.62 to 1.72 ms. The reason for such small errors for different starting points is that very often all RMS functions provided the same starting point for a given signal. In addition, there were cases where different starting points after some time of algorithm operation converged at one point, and the analysis comprised identical envelope fragments. Regardless of the envelope type, much better results were noted with additional MAA—the $\bar{\left|{\Delta T}_{i}\right|}$ ranged from 1.35 to 1.53 ms. The algorithms used for selection of the starting point and further correction MAA did not affect a level of the signal loss, whose value was around 0.4%.

### 3.2. Final Assessment

Section 3.1 presents the results regarding determination of control parameters for the algorithms D1 and D2. The final results of the algorithm performance were showed in relation to the original data from the fetal monitor. Table 5 presents final results obtained for FHR determination methods based on the Doppler ultrasound signal. For comparative purposes, the column FHR_CTG contains results obtained for a signal provided by a fetal monitor used in this study and thus calculated by its built-in US signal processing algorithms.

The mean value of differences $\bar{\Delta T_{i}}$ for both developed methods did not exceed 0.11 ms. In turn, standard deviation was 3.53 ms for D1 method and 2.14 ms for D2. The D2 method involves multiple AF calculation and segmentation of instantaneous measurements based on measured periodicity value. The method ensures that 95% of measurement errors do not exceed 4.28 ms. The mean $\bar{\left|{\Delta T}_{i}\right|}$ is 1.35 ms and is more than twice lower than for the original FHR_CTG signals determined inside a fetal monitor. The developed methods are characterized by a very low level of the signal loss, which for D2 is only 0.41%. Lack of signal loss for FHR_CTG results from wide AF window used, which is responsible for the measurement averaging, thus increasing the error value (Table 5).

A very important criterion for assessing the investigated methods are the errors of instantaneous FHR variability determination. Table 6 lists relative error values for the original FHR signal determined by US signal processing algorithms built-in a fetal monitor, the D1 method based on determination of subsequent FHR values every 250 ms and with duplicate measurements correction algorithm [65], as well as the D2 method in which time series of events is determined already at the stage of US signal analysis.

The values of all variability indices determined for the FHR_CTG signal are significantly lower (as negative values) than those obtained for the FHR_E reference signal. Errors of STV indices are in the range from −50.8% for the S_YEH index to as much as −91.3% for the S_HUE index. Such large errors are caused by duplicate measurements and the intervals averaging effect decreasing beat-to-beat variability.

This is confirmed by the results of D1 method, in which based on the developed algorithm of US signal analysis, instantaneous FHR values are calculated similarly to the fetal monitor (every 250 ms), and then corrected by detecting and removing duplicate measurements. This leads to significant error reduction, also for LTV indices. The D2 method provides the best representation of the real FHR signal variability, whose reference pattern comes from the signal derived from a direct FECG. For the most clinically relevant STV indices describing beat-to-beat variability, the error value ranged from −1.9% for S_ZUG to −10.1% for the S_HUE index.

## 4. Discussion

The paper proposed new methods for determining a fetal heart rate signal based on a detailed analysis of the raw Doppler ultrasound signal. The primary requirement for these methods was that the FHR signal should be in the form of time series of events reflecting subsequent cardiac cycles—T_i_ intervals.

Experimental studies were carried out based on simultaneously recorded Doppler ultrasound signal, original FHR_CTG signal calculated on its basis inside a fetal monitor, as well as the FHR_E signal determined from a direct fetal electrocardiogram (being the source of reference information). The effectiveness of the developed methods was evaluated on the basis of measurement accuracy of T_i_ intervals between successive heartbeats, combined with estimation of the resulting FHR signal loss. In turn, the second stage of assessment concerned the accuracy evaluation of the instantaneous FHR variability, based on dedicated indices describing short-term and long-term variability.

There are various ways to assess the quality of FHR signals from the outputs of popular ultrasound-based fetal monitors relative to the reference FECG. The comparison of signals in metrological aspect concerns both visual assessment of the FHR traces and estimation of statistical parameters describing the signals, as well as comparison of the synchronized measurements—T_i_ intervals. On the other hand, the verification of the ultrasound method in a clinical aspect consists in comparing diagnostically significant patterns determined on the FHR basis, such as: baseline, acceleration and deceleration patterns or indices describing instantaneous variability.

The first reports regarding the FHR signal recorded by an US method were published when fetal monitors became popular-in the seventies. Already in 1976, Lauersen [72] studied the differences between simultaneously recorded FHR signals via ultrasound and electrocardiography. The standard deviation for differences in corresponding measurements was as much as 9.8 bpm, mainly because of a lack of precise signal synchronization. Dawes [73] also omitted the synchronization problem—and when compared the FHR mean values representing one-hour signals—he obtained a correlation of 0.9. In addition, he assessed the FHR short-term variability using the S_DAW index, for which a correlation was 0.63. The difference between mean values of the S_DAW index: 2.3 ms for FECG and 4.6 ms for US, was statistically significant. Lawson [74] used the same method, but with a different fetal monitor. For FHR signals from the FECG channel, the mean S_DAW value was 2.1 ms, while for the US it was three times higher −6.2 ms. The obtained errors [72,73,74] resulted from using monitors with US channel of the so-called first generation, where T_i_ intervals were determined solely on the peak detection. In another work [75], Lawson has already used a monitor with a US channel of second generation, in which AF has already been applied to measure a periodicity. Furthermore, in this case, the difference was statistically significant, although much smaller and with the opposite trend—the S_DAW index was 1.29 ms for the US, which was 35% lower than its value of 1.98 ms for the FECG channel. The reason for underestimation was the autocorrelation function leading to averaging of T_i_ intervals.

Jezewski made a comprehensive assessment of the accuracy of FHR measurements [62], comparing 21,941 pairs of T_i_ intervals recorded simultaneously with US channel of fetal monitor MT-430 (Toitu) and direct fetal electrocardiography. Reference FECG was used to eliminate duplicate measurements, which after synchronization served as a clock signal for the FHR signal from a fetal monitor. This correction method has enabled a reliable assessment, but it was impossible to apply in clinical practice. For higher reliability of results, the FHR reference signal was determined by two independent algorithms from the FECG, and during final analysis the measurements for which the difference between them exceeded one millisecond were rejected. The mean error $\bar{\left|{\Delta T}_{i}\right|}$ between US and FECG was 2.98 ms. In turn, the errors of variability indices determination ranged from −2.0 to −9.3% for LTV and from −5.9 to as much as −39.5% for STV. No results were provided for uncorrected data from a fetal monitor, which would allow estimating the impact of duplicate measurements on errors of the instantaneous variability assessment.

Other studies concerned methods for determining FHR signals in the form of time series of events from measurements occurring every 250 ms and recorded with a classical fetal monitor [63,64,65]. Research conducted in [65] clearly showed that, in relation to the FECG the fetal heart rate signal containing duplicate measurements introduces errors, especially when determining the STV indices (from −51.5% to as much as −91.3%). Effective removal of duplicate measurements—conversion of the FHR signal to the form of time series of events—allowed to reduce these values (from −28.0% to −74.0%), which unfortunately still does not allow for reliable analysis of the FHR variability. This was confirmed by the results in [62], but although errors of the algorithm for duplicate measurements correction were eliminated by FECG signal timing, the errors of STV indices still had unacceptable values. The main reason was a use of too wide AF windows being responsible for measurements averaging. Therefore, only the development of more effective algorithms to analyze the raw US signal may improve the accuracy of FHR determination and thus the quantitative assessment of instantaneous variability.

Therefore, the authors developed two new methods for extraction of time series of events—the occurrences of fetal heartbeats. The first is D1 which is based on the duplicate measurements correction previously developed by the authors [65]. The obtained FHR signal and STV indices are much accurate than results obtained for the FHR_CTG signal from a fetal monitor. The mean error of interval measurements $\bar{\left|{\Delta T}_{i}\right|}$ was 1.91 ms, against 2.78 ms for the signal from a monitor output. For LTV indices, errors ranged from −0.1% to −1.4%. In contrast, for STV indices errors ranged from −2.1% to −13.3%. It is worth to note that, errors obtained for the original FHR_CTG signals were from −50.8% to −91.3%.

In the second D2 method, required events are results of segmentation of instantaneous FHR measurements, based on the analysis of measured values. This method provides a significantly lower interval measurement error of 1.35 ms. The increase in measurement accuracy does not occur at the expense of signal loss level equal to 0.41%. Errors of determining LTV indices using D2 were very similar to the D1 method and ranged from −0.1% to −1.3%. In the case of STV indices, errors were always lower than for D1 and ranged from −1.9% to −10.1%. The obtained results clearly confirm that the D2 method is the best in terms of both the accuracy of measurement and reliability of estimation of FHR instantaneous variability.

In general, the research carried out in the work covered a very wide range of issues, from an initial analysis of raw Doppler ultrasound signal after demodulation to the final measurement of FHR values. Therefore, the obtained results were influenced by many different control parameters. Some of them as: threshold of the AF peak amplitude defining the FHR signal loss and threshold deciding about the measurements support by an additional prediction function, were determined empirically basing on our previous experience [36,70]. Significantly larger group are parameters whose optimal values have been determined on the basis of results of research carried out as part of this study. These are: Doppler signal bandwidths, control parameters in determining the envelope signal, window parameters of the autocorrelation and prediction functions, as well as the width of the RMS window used in the final segmentation process. The process of determining all these parameters and evaluation of their impact on final results is documented in Section 3.1 Study of sensitivity.

The analyzed signal bandwidth has a significant impact on the results obtained. It should cover the range from 300 to 600 Hz corresponding to fetal heart valves. The use of short AF window (1 s) and measurement support with a trapezoidal prediction function turned out to be very important, which ensured high accuracy of T_i_ measurement and low signal loss in the resulting FHR. Nevertheless, for reliable assessment of indices describing beat-to-beat variability, a signal in the form of time series of events is required. In this case, better results were obtained using segmentation of instantaneous measurements F_j_.

As the final results showed, it was possible to obtain a high accuracy of T_i_ measurement and what is very important at a low level of FHR signal loss. However, we are far from claiming that the proposed algorithms are ready-to-use as completely reliable solutions. The work is primarily of a research nature, thanks to which it was shown that it is not the Doppler ultrasound signal itself (as representing less accurate mechanical activity of the fetal heart), that is the source of errors, but the methods of its analysis used.

The problem of determining the fetal heart rate in the form of time series of events for the US signal, which source was the second-generation HP 8040A monitor, was raised by Peters [66]. He applied a low-pass filter with a cutoff frequency of two hertz, and then the obtained markers representing the subsequent fetal heartbeats were used to separate fragments of an envelope signal, for which the Ti intervals were determined using AF. The 100-s signal was processed, and the results were referred to a direct FECG. The correlation coefficient between FHR signals was high and reached 0.977. Unfortunately, there was no assessment of the interval measurement accuracy, only a graph illustrating differences between the measurements for both methods against their means was provided.

A detailed assessment of an interval measurement error and STV indices determination based on an US signal analysis is provided in [67]. Referring to a simultaneously recorded FECG signal, in the US signal an AF window with the width of two full cardiac cycles was positioned. The tests concerned different values of window shift relative to an initial position, determined by the occurrence of the R wave. The minimum error $\bar{\left|{\Delta T}_{i}\right|}$ was 1.59 ms, while the minimum error of the S_DAW index was 7.12%. Unfortunately, the work has not any practical value because the US signal analysis requires to be supported by the FECG signal.

Jezewski also dealt with analysis of raw US signal to determine FHR in the form of time series of events [36]. The signals were recorded during labor using a prototype instrumentation, while the reference was a direct FECG signal. An AF window was used, whose width and step were changed adaptively depending on the last determined interval. The best results were obtained for a window width equal to twice the width of a previous interval, and for a step ensuring five measurements of each interval. The mean error $\bar{\left|{\Delta T}_{i}\right|}$ was 1.91 ms, with FHR signal loss of 1.6%. In addition, an error of −6.9% was determined for the S_HAA variability index.

Although investigations [36] were conducted using other database than in this study, in both cases a direct FECG was the reference method, and the number of analyzed intervals was similar—8945 against 7498 intervals examined by the authors. Therefore, the statistical significance of both studies represents similar level. This, in turn, allows to state that the results obtained in this work show higher accuracy of periodicity measurement and lower level of FHR signal loss. For the D2 method, the error in interval measuring was only 1.35 ms, with a signal loss of 0.41%, which confirms a high efficiency of the developed solution. However, lower interval error did not lead to decrease of the S_HAA variability index error, as it was −8.9%, while in [36] only −6.9%. Unfortunately, in [36] only S_HAA index was analyzed, whereas it is obvious that various indices react in different ways to the error in measuring the interval.

## 5. Conclusions

Using a popular fetal monitor based on the Doppler ultrasound technique, it is possible to analyze the fetal heart rate signal only in a classical approach, which consists in determining the FHR baseline for recognizing the patterns of tachycardia/bradycardia or acceleration/deceleration. However, it is almost impossible to reliably assess the instantaneous FHR variability that is a part of automated analysis carried out in computer-aided fetal monitoring system. The presented improvement was ensured by FHR signal correction consisting in removing of duplicate measurements. Thanks to it, the signal took the form of time series of events, as in electrocardiography. Unfortunately, from the point of view of fetal state assessment, errors of determining indices for instantaneous FHR variability remained at an unacceptable level. This means that another factor affects fetal monitors—the built-in algorithm for determining subsequent FHR values, focusing too much on being resistant to disturbances from fetal movements. Our research revealed that the Doppler ultrasound signal itself is not a source of errors. It is too long window in correlation methods that causes an effect of “averaging” several consecutive fetal heart cycles.

A comprehensive study of the Doppler US signal describing the mechanical activity of a fetal heart was carried out. The best results in terms of interval measurement accuracy and evaluation of instantaneous variability were obtained using the authors’ methods analyzing the raw Doppler ultrasound signal. The D2 method based on segmentation of instantaneous measurements particularly allows obtaining accuracy close to the electrocardiographic method. The results unambiguously confirm that it is possible to analyze the Doppler ultrasound signal in a way that allows effective determination of fetal heart rate in the form of time series of events—significantly improving the reliability of determining the indices for FHR instantaneous variability description.

## Figures and Tables

**Table 1:** Interval-measurement errors and FHR_U signal loss obtained for three US signal bandwidths and nine envelope determination methods when using a classical AF calculated every 250 ms for a window width of 1.5 s.

Envelope	$\bar{\left|\Delta T_{i}\right|}\;(\mathrm{ms})$			FHR_U Signal Loss (% )		
	100–300 Hz	300–600 Hz	100–600 Hz	100–300 Hz	300–600 Hz	100–600 Hz
Without filter	2.52 ^#^	2.12	2.16	3.11	3.43	2.23
Filter 25 Hz	2.27	1.99	2.07	2.43	2.55	1.71
Filter 50 Hz	2.26	**1.90 ^1^**	1.98	2.73	**2.81 ^1^**	1.87
Filter 75 Hz	2.26	1.92	1.97	2.80	3.00	1.96
Filter 100 Hz	2.26	1.92	**1.94 ^2^**	2.78	3.11	**2.01 ^2^**
Filter 150 Hz	2.30	1.95	1.97	2.81	3.18	2.05
Hilbert	2.35	1.99	2.01	2.65	3.12	2.02
Hilbert+MA11	2.25	1.90	**1.93 ^3^**	2.55	2.96	**1.87 ^3^**
Hilbert+MA21	2.26	**1.90 ^4^**	1.99	2.45	**2.56 ^4^**	1.97

^#^ mean value ^1–4^ four parameter settings to be used in further experiments.

**Table 2:** Interval measurement errors and signal loss level in the FHR_U for two AF repetition cycles K (25 and 250 ms), using the PF with different window widths S or without PF. Four combinations of US signal bandwidths and four envelopes were considered.

K (ms)	S (ms)	$\bar{\left|\Delta T_{i}\right|}\;(\mathrm{ms})$				FHR_U Signal Loss (% )			
		100–600 Filter 100	100–600 Hilbert	300–600 Filter 50	300–600 Hilbert	100–600 Filter 100	100–600 Hilbert	300–600 Filter 50	300–600 Hilbert
**250**	–	1.95 ^#^	1.94	1.91	1.91	2.01	1.87	2.82	2.56
	125	3.17	3.02	3.04	3.03	6.97	6.74	6.13	6.84
	250	2.14	2.12	2.08	2.07	1.17	1.07	0.75	0.88
	500	2.02	2.02	**1.99 ^1^**	**2.00 ^2^**	0.78	0.79	**0.56 ^1^**	**0.56 ^2^**
25	–	1.96	1.94	1.89	1.90	1.92	1.84	2.81	2.43
	125	3.00	2.97	3.00	2.91	6.28	5.98	6.39	5.97
	250	2.11	2.1	2.09	2.07	1.39	1.28	0.91	0.91
	500	2.02	2.02	**1.98 ^3^**	**1.96 ^4^**	0.8	0.79	**0.57 ^3^**	**0.59 ^4^**

^#^ mean value ^1–4^ four parameter settings to be used in further experiments.

**Table 3:** Interval measurement errors and the FHR_U signal loss for different AF window widths D and various combinations of other control parameters.

K (ms)	D (s)	$\bar{\left|\Delta T_{i}\right|}\;(\mathrm{ms})$				FHR_U Signal Loss (% )			
		Without PF		PF (S = 500 ms)		Without PF		PF (S = 500 ms)	
		Filter 50	Hilbert	Filter 50	Hilbert	Filter 50	Hilbert	Filter 50	Hilbert
**250**	0.8	1.37 ^#^	1.41	1.43	1.45	22.29	22.00	1.82	1.74
	1	1.21	1.24	1.24	1.26	3.47	3.46	0.57	0.55
	1.5	1.91	1.91	1.99	2.00	2.82	2.56	0.56	0.56
	2	2.44	2.42	2.54	2.51	2.42	2.16	0.69	0.67
	3	3.38	3.36	3.50	3.48	2.62	2.36	0.92	0.90
25	0.8	1.46	1.45	1.34	1.35	20.53	20.52	0.97	0.94
	1	1.20	1.22	**1.24 ^1^**	**1.25 ^2^**	3.06	3.08	**0.48 ^1^**	**0.47 ^2^**
	1.5	1.89	1.90	1.98	1.96	2.81	2.43	0.57	0.59
	2	2.42	2.41	2.55	2.52	2.47	2.20	0.69	0.71
	3	3.36	3.34	3.50	3.47	2.47	2.34	0.93	0.92

^#^ mean value ^1–2^ two parameter settings to be used in further experiments.

**Table 4:** Interval measurements errors and the FHR_U signal loss for different starting points, with and without additional matching algorithm MAA.

RMS Window Width (ms)	$\bar{\left|\Delta T_{i}\right|}\;(\mathrm{ms})$				FHR_U Signal Loss (% )			
	Without MAA		MAA		Without MAA		MAA	
	Filter 50	Hilbert	Filter 50	Hilbert	Filter 50	Hilbert	Filter 50	Hilbert
**–**	1.62 ^#^	1.63	1.44	1.45	0.43	0.43	0.43	0.44
**100**	1.64	1.67	1.43	1.45	0.38	0.36	0.40	0.44
**200**	1.67	1.70	1.48	1.43	0.38	0.42	0.40	0.43
**250**	1.66	1.69	1.53	1.46	0.41	0.41	0.39	0.43
**500**	1.68	1.61	1.45	**1.35 ^x^**	0.47	0.43	0.44	**0.41 ^x^**
**1000**	1.69	1.72	1.48	1.44	0.41	0.43	0.41	0.42

^#^ mean value ^x^ minimal T_i_ error value determining the optimal values of control parameter.

**Table 5:** Final results obtained for various methods for FHR_U signal determination based on a raw US signal in relation to the reference FHR_E from direct FECG.

Parameter	FHR Determination Method		
	FHR_CTG	FHR_U D1	FHR_U D2
$\bar{\Delta T_{i}}$ **(ms)**	0.13	0.11	**0.04**
$SD\_\Delta T_{i}\;$ **(ms)**	4.01	3.53	**2.14**
$\bar{\left|\Delta T_{i}\right|}$ **(ms)**	2.78	1.91	**1.35**
**FHR signal loss (%)**	0.0	0.47	**0.41**

**Table 6:** Relative errors δ for STV and LTV variability indices, determined for original signals FHR_CTG from a fetal monitor output, and for FHR_U from the new Doppler ultrasound signal analysis methods, in relation to the FHR_E reference signal from FECG.

δ (%)	FHR Determination Method		
	FHR_CTG	FHR_U D1	FHR_U D2
**STV**			
δS_DAW	−66.2 ± 7.99 ^#^	−6.4 ± 6.25	**−5.0 ± 4.75**
δS_YEH	−50.8 ± 11.07	−3.0 ± 7.69	**−2.4 ± 6.78**
δS_HAA	−80.0 ± 7.27	−13.3 ± 12.32	**−8.9 ± 12.02**
δS_ZUG	−51.5 ± 9.28	−2.1 ± 9.09	**−1.9 ± 8.15**
δS_HUE	−91.3 ± 8.73	−11.9 ± 16.07	**−10.1 ± 12.00**
δS_DAL	−65.5 ± 7.59	−6.9 ± 6.25	**−5.4 ± 4.72**
**LTV**			
δL_DAW	−10.3 ± 9.00	−0.7 ± 6.47	**−0.1 ± 5.76**
δL_YEH	−6.3 ± 6.78	−0.3 ± 2.79	**−0.1 ± 2.36**
δL_HAA	−2.3 ± 11.16	−1.4 ± 6.89	**−0.9 ± 5.09**
δL_ZUG	−6.7 ± 7.26	−0.2 ± 2.50	**−0.1 ± 2.19**
δL_HUE	9.80 ± 16.95	−1.3 ± 10.22	**−1.3 ± 8.04**
δL_DAL	−5.9 ± 6.93	−0.1 ± 2.87	**−0.1 ± 2.14**

^#^ mean value ± SD.

## References

[1] Horoba K., Wrobel J., Jezewski J., Kupka T., Roj D., Jezewski M. (2016). Automated detection of uterine contractions in tocography signals–comparison of algorithms. Biocybern. Biomed. Eng..

[2] Jezewski J., Matonia A., Czabanski R., Horoba K., Kupka T., Burduk R., Jackowski K., Kurzynski M., Wozniak M., Zolnierek A. (2013). Classification of Uterine Electrical Activity Patterns for Early Detection of Preterm Birth. Computer Recognition Systems 8–CORES 2013.

[3] Jezewski J., Wrobel J., Horoba K., Gacek A., Sikora J. Fetal heart rate variability: Clinical experts versus computerized system interpretation. Proceedings of the 24th International Conference of IEEE Engineering in Medicine and Biology Society.

[4] Horoba K., Jezewski J., Matonia A., Wrobel J., Czabanski R., Jezewski M. (2016). Early predicting a risk of preterm labour by analysis of antepartum electrohysterograhic signals. Biocybern. Biomed. Eng..

[5] Sikora J., Matonia A., Czabanski R., Horoba K., Jezewski J., Kupka T. (2011). Recognition of Premature Threatening Labour Symptoms from Bioelectrical Uterine Activity Signals. Arch. Perinat Med..

[6] Kording F., Schoennagel B., Lund G., Ueberle F., Jung C., Adam G., Yamamura J. (2014). Doppler ultrasound compared with electrocardiogram and pulse oximetry cardiac triggering: A pilot study. Magn. Reson. Med..

[7] Jaros R., Martinek R., Kahankova R. (2018). Non-Adaptive Methods for Fetal ECG Signal Processing: A Review and Appraisal. Sensors.

[8] Van Scheepen J.A.M., Koster M.P.H., Vasak B., Redman C., Franx A., Georgieva A. (2016). Effect of signal acquisition method on the fetal heart rate analysis with phase rectified signal averaging. Physiol. Meas..

[9] Jezewski J., Horoba K., Roj D., Wrobel J., Kupka T., Matonia A. (2016). Evaluating the fetal heart rate baseline estimation algorithms by their influence on detection of clinically important patterns. Biocybern. Biomed. Eng..

[10] Jezewski J., Horoba K., Matonia A., Gacek A., Bernyś M. A new approach to cardiotocographic fetal monitoring based on analysis of bioelectrical signals. Proceedings of the 25th International Conference of IEEE Engineering in Medicine and Biology Society.

[11] Jezewski J., Wrobel J., Matonia A., Horoba K., Martinek R., Kupka T., Jezewski M. (2017). Is abdominal fetal electrocardiography an alternative to Doppler ultrasound for FHR variability evaluation?. Front. Physiol..

[12] Kahankova R., Jezewski J., Nedoma J., Jezewski M., Fajkus M., Kawala-Janik A., Wen H., Martinek R. (2017). Influence of gestation age on the performance of adaptive systems for fetal ECG extraction. Adv. Electr. Electron. Eng..

[13] Jezewski J., Matonia A., Kupka T., Roj D., Czabański R. (2012). Determination of the fetal heart rate from abdominal signals: Evaluation of beat-to-beat accuracy in relation to the direct fetal electrocardiogram. Biomed. Eng..

[14] Kahankova R., Jaros R., Martinek R., Jezewski J., Wen H., Jezewski M., Kawala-Janik A. (2017). Non-Adaptive Methods of Fetal ECG Signal Processing. Adv. Electr. Electron. Eng..

[15] Martinek R., Kahankova R., Jezewski J., Jaros R., Mohylova J., Fajkus M., Nedoma J., Janku P., Nazeran H. (2018). Comparative Effectiveness of ICA and PCA in Extraction of fECG from aECG Signals: Towards Multichannel Non-Invasive fHR Monitoring. Front. Physiol..

[16] Docker M.F. (1993). Doppler ultrasound monitoring technology. Br. J. Obstet. Gynaecol..

[17] Khandoker A.H., Kimura Y., Ito T., Palaniswami M. (2007). Non-invasive determination of electromechanical time intervals of cardiac cycle using abdominal ECG and Doppler ultrasound signals from fetal hearts. Comput. Cardiol..

[18] Abdulhay E.W., Oweis R.J., Alhaddad A.M., Sublaban F.N., Radwan M.A., Almasaeed H.M. (2014). Non-Invasive Fetal Heart Rate Monitoring Techniques: Review Article. Biomed. Sci. Eng..

[19] Alnuaimi S., Jimaa S., Khandoker A.H. (2017). A Review of Fetal cardiac Doppler Signal Processing for Screening Foetal Well Being. Front. Bioeng. Biotechnol..

[20] Kording F., Schoennagel B., Tavares de Sousa M., Fehrs K., Adam G., Yamamura J., Ruprecht C. (2018). Evaluation of a Portable Doppler Ultrasound Gating Device for Fetal Cardiac MR Imaging: Initial Results at 1.5T and 3T. Magn. Reson. Med. Sci..

[21] Mert A., Sezdi M., Akan A. (2015). A test and simulation device for Doppler-based fetal heart rate monitoring. Turk. J. Electr. Eng. Comput. Sci..

[22] Karjadi M., Salahuddin N.S., Wibowo E.P., Afandi H. (2016). Digital Filter Design of Infinite Impulse Response (IIR) Infrasound to Detect Fetal Heart Rate. Int. J. Eng. Res. Sci..

[23] Kupka T., Jeżewski J., Matonia A., Horoba K., Wróbel J. Timing events in Doppler ultrasound signal of fetal heart activity. Proceedings of the 26th International Conference of IEEE Engineering in Medicine and Biology Society.

[24] Voicu I., Girault J.M., Roussel C., Decock A., Kouame D. (2010). Robust estimation of fetal heart rate from US Doppler signals. Phys. Procedia.

[25] Marzbanrad F., Kimura Y., Funamoto K., Sugibayashi R., Endo M., Ito T., Palaniswami M., Khandoker A.H. (2014). Automated Estimation of Fetal Cardiac Timing Events From Doppler Ultrasound Signal Using Hybrid Models. IEEE J. Biomed. Health Inform..

[26] Marzbanrad F., Kimura Y., Funamoto K., Oshio S., Endo M., Sato N., Palaniswami M., Khandoker A.H. (2016). Model-Based Estimation of Aortic and Mitral Valves Opening and Closing Timings in Developing Human Fetuses. IEEE J. Biomed. Health Inform..

[27] Marzbanrad F., Stroux L., Clifford G.D. (2018). Cardiotocography and beyond: A review of one-dimensional Doppler ultrasound application in fetal monitoring. Physiol. Meas..

[28] Khandoker A., Kimura Y., Ito T., Sato N., Okamura K., Palaniswami M. (2009). Antepartum non-invasive evaluation of opening and closing timings of the cardiac valves in fetal cardiac cycle. Med. Biol. Eng. Comput..

[29] Lee C.S., Masek M., Lam C.P., Tan K.T. Towards Higher Accuracy and Better Noise-Tolerance for Fetal Heart Rate Monitoring using Doppler Ultrasound. Proceedings of the IEEE Region 10 Conference (TENCON).

[30] Voicu I., Menigot S., Kouame D., Girault J.M. (2014). New estimators and guidelines for better use of fetal heart rate estimators with doppler ultrasound devices. Comput. Math. Method Med..

[31] Shakespeare S.A., Crowe J.A., Hayes-Gill B.R., Bhogal K., James D.K. (2001). The information content of Doppler ultrasound signals from the fetal heart. Med. Biol. Eng. Comput..

[32] Wrobel J., Roj D., Jezewski J., Horoba K., Kupka T., Jezewski M. (2015). Evaluation of the robustness of fetal heart rate variability measures to low signal quality. J. Med. Imaging Health Inform..

[33] Hamelmann P., Mischi M., Kolen A.F., van Laar J.O.E.H., Vulings R., Bergmans J.W.M. (2019). Fetal Heart Rate Monitoring Implemented by Dynamic Adaptation of Transmission Power of a Flexible Ultrasound Transducer Array. Sensors.

[34] Vlachos M., Yu P., Castelli V. On Periodicity Detection and Structural Periodic Similarity. Proceedings of the 5th SIAM International Conference on Data Mining.

[35] Hamelmann P., Vulings R., Kolen A.F., Bergmans J.W.M., van Laar J.O.E.H., Tortoli P., Mischi M. (2019). Doppler ultrasound yechnology for fetal heart rate monitoring: A review. IEEE Trans. Ultrason. Ferroelectr..

[36] Jezewski J., Roj D., Wróbel J., Horoba K. (2011). A nowel technique for fetal heart rate estimation from Doppler ultrasound signal. Biomed. Eng. Online.

[37] Hamelmann P., Vullings R., Mischi M., Kolen A.F., Schmitt L., Bergmans J.W.M. (2019). An Extended Kalman Filter for Fetal Heart Location Estimation During Doppler-Based Heart Rate Monitoring. IEEE Trans. Instrum. Meas..

[38] Valderrama C.E., Marzbanrad F., Stroux L., Clifford G.D. (2017). Template-based Quality Assessment of the Doppler Ultrasound Signal for Fetal Monitoring. Front. Physiol..

[39] Divon M.Y. (1985). Autocorrelation techniques in fetal monitoring. Am. J. Obstet. Gynecol..

[40] Fukushima T., Flores C.A. (1985). Limitations of autocorrelation in fetal heart rate monitoring. Am. J. Obstet. Gynecol..

[41] Murrills A.J., Wilmshurst T.H., Wheeler T. (1986). Antenatal measurement of beat-to-beat fetal heart rate variation: Accuracy of the Hewlett-Packard ultrasound autocorrelation technique. Fetal Physiol. Meas..

[42] Taylor J., Paull C.J., Hayes-Gill B.R., Crowe J.A. (1997). Data compression of fetal Doppler ultrasound audio signals using zero-crossing analysis. Med. Eng. Phys..

[43] Spilka J., Chudacek V., Bursa M., Zach L., Huptych M., Lhotska L., Janku P., Hruban L. (2012). Stability of Variability Features Computed from Fetal Heart Rate with Artificially Infused Missing Data. Comput. Cardiol..

[44] Zhang L., Huang M.J., Wang H.J. (2019). A Novel Technique for Fetal Heart Rate Estimation Based on Ensemble Learning. Mod. Appl. Sci..

[45] Tuck D.L. (1982). Improvement in Doppler ultrasound human foetal heart rate records by signal correlation. Med. Biol. Eng. Comput..

[46] Romano M., Bifulco P., Ruffo M., Improta G., Clemente F., Cesarelli M. (2016). Software for computerised analysis of cardiotocographic traces. Comput. Meth. Prog. Biomed..

[47] Romano M., Iuppariello L., Ponsiglione A.M., Improta G., Bifulco P., Cesarelli M. (2016). Frequency and Time Domain Analysis of Foetal Heart Rate Variability with Traditional Indexes: A Critical Survey. Comput. Math. Method. Med..

[48] Wrobel J., Kupka T., Horoba K., Matonia A., Roj D., Jezewski J. (2015). Recognition of fetal movements–automated detection from Doppler ultrasound signals compared to maternal perception. J. Med. Imaging Health Inform..

[49] Czabanski R., Jezewski J., Wrobel J., Sikora J., Jezewski M. (2013). Application of fuzzy inference system for classification of fetal heart rate tracings in relation to neonatal outcome. Gin. Pol..

[50] Jezewski M., Czabanski R., Horoba K., Leski J.M. (2016). Clustering with pairs of prototypes to support automated assessment of the fetal state. Appl. Artif. Intell..

[51] Jezewski M., Leski J.M., Czabanski R., Gruca A., Brachman A., Kozielski S., Czachorski T. (2016). Classification based on incremental fuzzy (1+p) -means clustering. Man-Machine Interactions 4.

[52] Importa G., Romano M., Ponsiglione A., Bifulco P., Faiella G., Cesarelli M. (2014). Computerized Cardiotocography: A Software to Generate Synthetic Signals. J. Health Med. Inf..

[53] Mantel R., Ververs I., Colenbrander G.J., van Geijn H.P., van Geijn H.P., Copray F.J.A. (1994). Automated antepartum baseline FHR determination and detection of accelerations and decelerations. A Critical Appraisal of Fetal Surveillance.

[54] Jezewski J., Pawlak A., Horoba K., Wrobel J., Czabanski R., Jezewski M. (2016). Selected Design Issues of the Medical Cyber-Physical System for Telemonitoring Pregnancy at Home. Microprocess. Microsyst..

[55] Wrobel J., Jezewski J., Horoba K., Pawlak A., Czabanski R., Jezewski M., Porwik P. (2015). Medical cyber-physical system for home telecare of high-risk pregnancy–design challenges and requirements. J. Med. Imaging Health Inform..

[56] Wrobel J., Matonia A., Horoba K., Jezewski J., Czabanski R., Pawlak A., Porwik P. (2015). Pregnancy telemonitoring with smart control of algorithms for signal analysis. J. Med. Imaging Health Inform..

[57] Sankhe M.S., Desai K.D., Gadam M.A. (2016). Estimate of Fetal Autonomic State by Time Spectral and Nonlinear Analysis of Fetal Heart Rate Variability. Int. J. Comput. Inf. Syst..

[58] Kubo T., Inaba J., Shigemitsu S., Akatsuka T. (1987). Fetal heart variability indices and the accuracy of variability measurements. Am. J. Perinat.

[59] Cesarelli M., Romano M., Bifulco P. (2009). Comparison of short term variability indexes in cardiotocographic foetal monitoring. Comput. Biol. Med..

[60] Goncalves H., Chaves J., Costa A., Ayres-de-Campos D., Bernardes J. (2015). Comparison of the effect of different sampling modes on computer analysis of cardiotocograms. Comput. Biol. Med..

[61] Goncalves H., Costa A., Ayres-de-Campos D., Costa-Santos C., Rocha A.P., Bernardes J. (2013). Comparison of real beat-to-beat signals with commercially available 4 Hz sampling on the evaluation of foetal heart rate variability, available 4 Hz sampling on the evaluation of foetal heart rate variability. Med. Biol. Eng. Comput..

[62] Jezewski J., Wróbel J., Horoba K. (2006). Comparison of Doppler ultrasound and direct electrocardiography acquisition techniques for quantification of fetal heart variability. IEEE Trans. Biomed. Eng..

[63] Cesarelli M., Romano M., Bifulco P., Fedele F., Bracale M. (2007). An algorithm for the recovery of fetal heart rate series from CTG data. Comput. Biol. Med..

[64] Jezewski J., Kupka T., Horoba K. (2008). Extraction of Fetal Heart Rate Signal as Time Event Series from Evenly Sampled Data Acquired Using Doppler Ultrasound Technique. IEEE Trans. Biomed. Eng..

[65] Kupka T., Matonia A., Jezewski M., Horoba K., Wrobel J., Jezewski J. (2020). Coping with limitations of fetal monitoring instrumentation to improve heart rhythm variability assessment. Biocybern. Biomed. Eng..

[66] Peters C.H.L., ten Broeke E.D., Andriessen P., Vermeulen B., Berendsen R.C.M., Wijn P.F.F., Oei S.G. (2004). Beat-to-beat detection of fetal heart rate: Doppler ultrasound cardiotocography compared to direct ECG cardiotocography in time and frequency domain. Physiol. Meas..

[67] Roj D., Kupka T., Czabański R., Pander T., Jeżewski J. Improvement in fetal heart periodicity measurement using Doppler ultrasound signal. Proceedings of the 5th European Conference of the International Federation for Medical and Biological Engineering.

[68] Al-Angari H.M., Kimura Y., Hadjileontiadis J., Khandoker A.H. (2017). A Hybrid EMD-Kurtosis Method for Estimating Fetal Heart Rate from Continuous Doppler Signals. Front. Physiol..

[69] van Geijn H.P. (1980). Analysis of heart rate and beat-to-beat variability: Interval difference index. Am. J. Obstet. Gynecol..

[70] Jezewski M., Czabanski R., Wrobel J., Horoba K. (2010). Analysis of Extracted Cardiotocographic Signal Reatures to Improve Automated Prediction of Fetal Outcome. Biocybern. Biomed. Eng..

[71] Frigo G., Giorgi G. Comparative Evaluation of On-Line Missing Data Regression Techniques in Intrapartum FHR Measurements. Proceedings of the IEEE IMTC Conference.

[72] Lauersen N.H., Hochberg H.M., George M.E.D. (1976). Evaluation of the accuracy of a new ultrasonic fetal heart rate monitor. Am. J. Obstet. Gynecol..

[73] Dawes G.S., Visser G.H.A., Goodman J.D.S., Redman C.W.G. (1981). Numerical analysis of the human fetal heart rate: The quality of ultrasound records. Am. J. Obstet. Gynecol..

[74] Lawson G.W., Dawes G.S., Redman C.W.G. (1982). A comparison of two fetal heart rate ultrasound detector systems. Am. J. Obstet. Gynecol..

[75] Lawson G.W., Belcher R., Dawes G.S., Redman C.W.G. (1983). A comparison of ultrasound (with autocorrelation) and direct electrocardiogram fetal heart rate detector systems. Am. J. Obstet. Gynecol..

